# Supplementary material for: Splice-Junction-Based Mapping of Alternative Isoforms in the Human Proteome
Source: Cell Rep. Author manuscript; Available in PMC 2020 Jan 15. (PMC6961840; doi:10.1016/j.celrep.2019.11.026)
Supplement: 2 [file NIHMS1546469-supplement-2.zip › DF1/PXD009737/TestisTrypsin/TestisTrypsin_11_RHOT2_LSDQDLDQALSDEELNAFQQK.pdf]

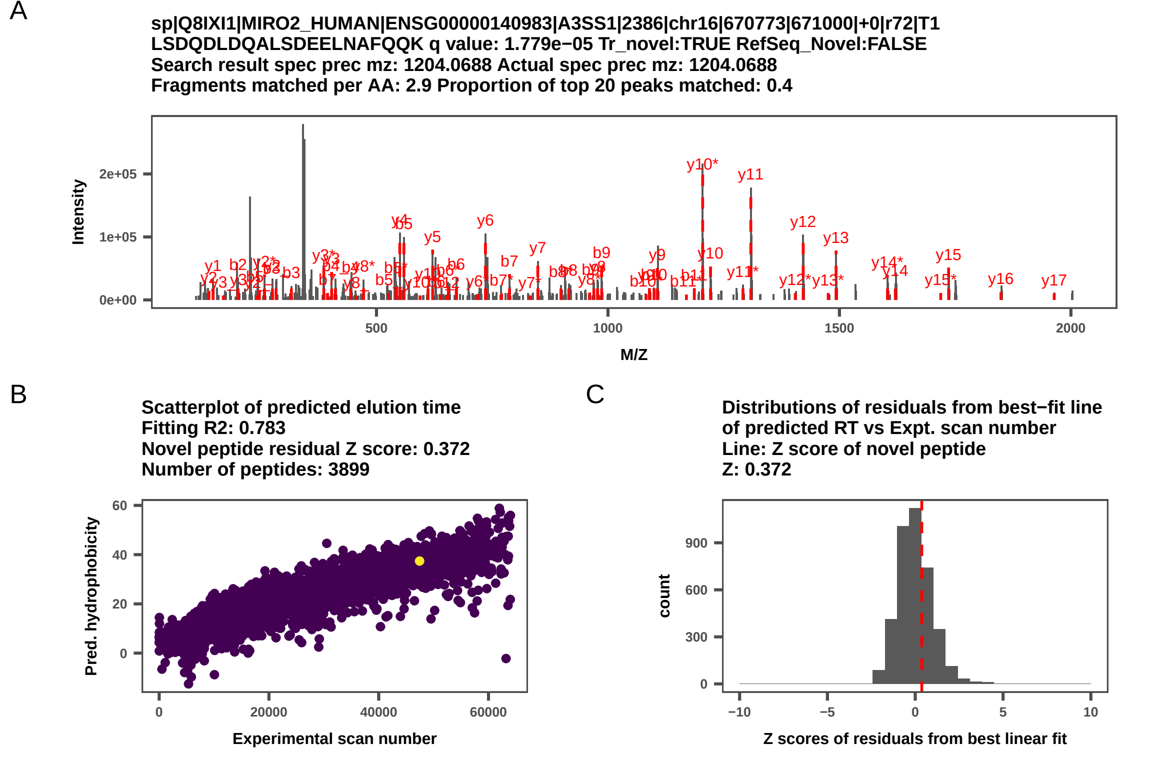
A
sp|Q8IXI1|MIRO2_HUMAN|ENSG00000140983|A3SS1|2386|chr16|670773|671000|+0|r72|T1
LSDQDLDQALSDEELNAFQQK q value: 1.779e−05 Tr_novel:TRUE RefSeq_Novel:FALSE
Search result spec prec mz: 1204.0688 Actual spec prec mz: 1204.0688
Fragments matched per AA: 2.9 Proportion of top 20 peaks matched: 0.4
Intensity
2e+05
1e+05
0e+00
M/Z
500
1000
1500
2000
y10*
y11
y12
y13
y6
y5
y4
y7
b7
b9
y9
y10
y11*
y12*
y13*
y14*
y14
y15
y15*
y16
y17
B
Scatterplot of predicted elution time
Fitting R2: 0.783
Novel peptide residual Z score: 0.372
Number of peptides: 3899
Pred. hydrophobicity
60
40
20
0
0
20000
40000
60000
Experimental scan number
C
Distributions of residuals from best−fit line
of predicted RT vs Expt. scan number
Line: Z score of novel peptide
Z: 0.372
count
900
600
300
0
−10
−5
0
5
10
Z scores of residuals from best linear fit
